# Block of NMDA receptor channels by endogenous neurosteroids: implications for the agonist induced conformational states of the channel vestibule

**DOI:** 10.1038/srep10935

**Published:** 2015-06-18

**Authors:** Vojtech Vyklicky, Barbora Krausova, Jiri Cerny, Ales Balik, Martin Zapotocky, Marian Novotny, Katarina Lichnerova, Tereza Smejkalova, Martina Kaniakova, Miloslav Korinek, Milos Petrovic, Petr Kacer, Martin Horak, Hana Chodounska, Ladislav Vyklicky

**Affiliations:** 1 Institute of Physiology CAS, Videnska 1083, 142 20 Prague 4, Czech Republic; 2 Charles University in Prague, Faculty of Science, Albertov 6, 128 43 Prague 2, Czech Republic; 3 Institute of Chemical Technology—Prague, Technicka 5, 166 28 Prague, Czech Republic; 4 Institute of Organic Chemistry and Biochemistry CAS, Flemingovo nam. 2, 166 10 Prague 2, Czech Republic; 5 School of Pharmacy and Biomedical Sciences, University of Central Lancashire, Preston, PR1 2HE, UK; 6 Institute of Medical Physiology, School of Medicine, University of Belgrade, Visegradska 26/II, 11000 Beograd, Srbija

## Abstract

N-methyl-D-aspartate receptors (NMDARs) mediate synaptic plasticity, and their dysfunction is implicated in multiple brain disorders. NMDARs can be allosterically modulated by numerous compounds, including endogenous neurosteroid pregnanolone sulfate. Here, we identify the molecular basis of the use-dependent and voltage-independent inhibitory effect of neurosteroids on NMDAR responses. The site of action is located at the extracellular vestibule of the receptor’s ion channel pore and is accessible after receptor activation. Mutations in the extracellular vestibule in the SYTANLAAF motif disrupt the inhibitory effect of negatively charged steroids. In contrast, positively charged steroids inhibit mutated NMDAR responses in a voltage-dependent manner. These results, in combination with molecular modeling, characterize structure details of the open configuration of the NMDAR channel. Our results provide a unique opportunity for the development of new therapeutic neurosteroid-based ligands to treat diseases associated with dysfunction of the glutamate system.

*N*-methyl-D-aspartate receptors (NMDARs) are ligand-gated ion channels permeable to calcium that play important roles in synaptogenesis, synaptic plasticity, learning, and memory^1^,^2^. However, abnormal activation of NMDARs is thought to mediate neuronal degeneration^3^,^4^.

Among the powerful allosteric modulators of NMDARs are neurosteroids that have been shown to induce potentiation, inhibition or mixed effects^5^. These steroids represent a broad class of endogenous compounds whose site of synthesis and action is in the nervous system itself. They play multiple biological roles both during embryogenesis and in adults^6^. Even though it is difficult to interpret their behavioral effects based on affinities to specific neurotransmitter receptors, it is plausible to speculate that neuroprotective activity and improvements of behavioral outcomes after hippocampal and spinal cord lesions are related to the direct action of endogenous neurosteroid 20-oxo-5β-pregnan-3α-yl sulfate (pregnanolone sulfate; PA-S) and its structural analogs at NMDARs^7^,^8^,^9^,^10^. Surprisingly, even at high neuroprotective concentrations, they lack psychomimetic behavioral effects typical for use- and voltage-dependent NMDAR channel inhibitors^9^,^11^.

Kinetic, pharmacological and optical experiments showed that the steroid inhibition was not typical for a drug-receptor interaction in an aqueous solution compatible with the hypothesis that steroid accumulation in the plasma membrane is the route by which it accesses a binding site on the NMDAR^12^. Interestingly, the action of PA-S has some characteristics similar to the action of voltage-dependent NMDAR channel inhibitors. For example, the access of PA-S to its site of action is conditioned by the activation of NMDARs by agonists (use-dependent allosteric inhibition). However, in contrast to voltage-dependent inhibitors, the action of both the positively and negatively charged steroids at the NMDAR is voltage-independent^10^,^12^,^13^,^14^,^15^.

Here, we have used electrophysiological and molecular biology techniques in combination with molecular modeling to analyze molecular mechanisms of steroid action at NMDARs. Our results suggest that neurosteroids bind in the extracellular vestibule of the NMDAR channel to prevent the permeation of small mono and divalent ions. In addition, the model of the NMDAR channel opening provides an explanation for the different contributions of GluN1 and GluN2B subunits to the inhibition by the steroid.

## Results

### Steroid inhibition of NMDARs is use-dependent

**Figure 1:**
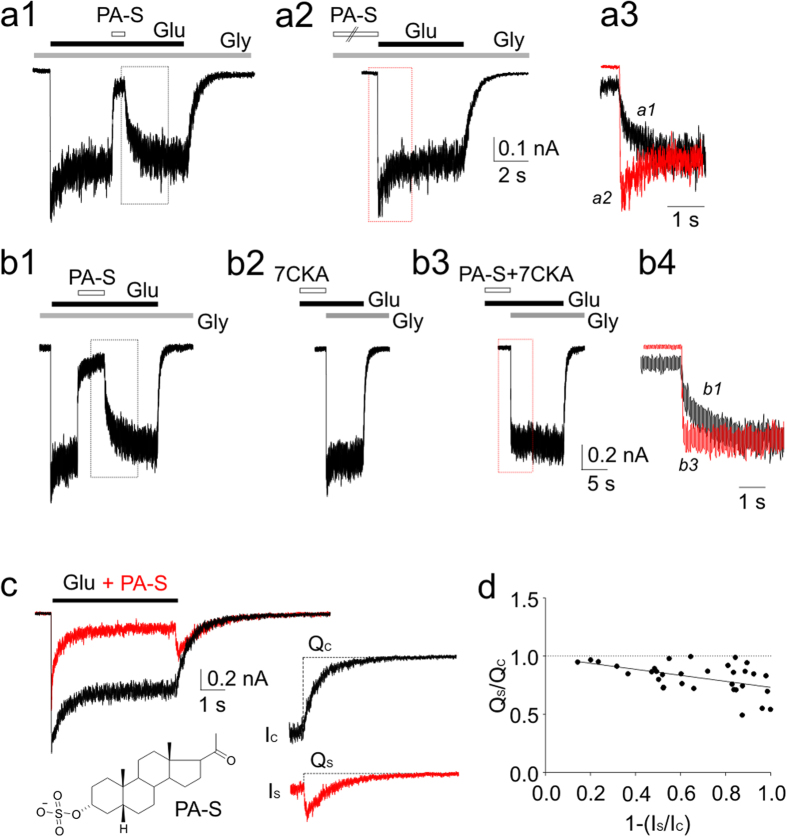
Neurosteroid-induced inhibition is use-dependent. (**a1**) Current response of GluN1/GluN2B receptors to glutamate (1 mM) (in the presence of 10 μM glycine) was inhibited by PA-S (150 μM) with slow off kinetics (left). (**a2**) The onset of the response to glutamate was not affected by 5 s pre-application of PA-S in the presence of 10 μM glycine (middle). (**a3**) The off responses to PA-S washout following its application in the presence of glutamate and glycine (black trace; dotted box in a1) and of glycine only (red trace; dotted box in a2) are shown overlaid. (**b1**) Control response to PA-S made in the presence of glutamate and glycine. (**b2**) Response to glutamate (1 mM) and glycine (30 μM) made following glutamate (1 mM) and 7-CKA (100 μM) co-application in the absence of added glycine. (**b3**) Response to glycine and glutamate made following PA-S pre-application for 5 s in the presence of glutamate, 7-CKA, and absence of added glycine. (**b4**) The off responses to PA-S washout following its application in the presence of glutamate and glycine (black trace; dotted box in b1) and glutamate, 7-CKA, and absence of added glycine (red trace; dotted box in b3) are shown overlaid. (**c**) Superposition of a control response to glutamate (1 mM) (black) and glutamate response made in the presence of PA-S (100 μM) (red). Inset shows the structure of PA-S. (**d**) Graph of the control-normalized charge transfer during the after-current (*Qs/Qc*) and the extent of the stationary current inhibition (1–(*I*_S_/*I*_C_)). The after-current was recorded after glutamate and PA-S (10, 30, 50, 100, 150, 300 μM) co-application. Data were fitted with linear regression with a slope of –0.26 (*n* = 7).

To investigate the dependence of PA-S inhibition on NMDAR activation, cDNA encoding for the GluN1 and GluN2B subunits were co-transfected into HEK293 cells. Figure 1a shows control responses of the transfected cells to a fast application of glutamate (1 mM) recorded in the presence of 10 μM glycine and no added Mg^2+^. The responses to a co-application of 150 μM PA-S and glutamate made after the onset of the response to glutamate were inhibited with a fast onset (τ_f_ = 30 ± 2 ms (A_f_ = 75 ± 3%); τ_s_ = 579 ± 92 ms; *n* = 6) and recovered from inhibition with a slow offset (τ_f_ = 82 ± 14 ms (A_f_ = 45 ± 9%); τ_s_ = 536 ± 79 ms; *n* = 6). The time constant of the slow rate of recovery from inhibition was 902 ± 201 ms (*n* = 5) for glutamate and steroid co-application for 30 s. The onset of the response to glutamate application (10–90%, 15.5 ± 2.9 ms, *n* = 5) was not significantly different from that recorded immediately after 30 s PA-S (150 μM) pre-application in the absence of glutamate (18.4 ± 1.6 ms, *n* = 5; P = 0.306). These results show the use-dependent action of PA-S at the NMDAR, indicating that in the presence of only glycine, PA-S cannot bind; however, after binding both glutamate and glycine, the NMDAR reaches conformational state(s) that allow steroid binding.

Similar results were observed for responses induced by glycine (30 μM) application made in the continuous presence of glutamate. To avoid activation of NMDARs in the presence of 1 mM glutamate and no added glycine by its residual concentration in the extracellular solution (ECS), 7-Cl-kynurenic acid (7-CKA; 100 μM) was used in the control and during PA-S pre-application (Fig. 1b). The onset of responses to glycine application made in the continuous presence of glutamate (1 mM) and immediately after 7-CKA pre-application was fit by a single exponential function (τ = 21 ± 1 ms; *n* = 5) (Fig. 1b). Response to glycine made immediately after 5 s 150 μM PA-S pre-application in the continuous presence of glutamate (1 mM) was fit by a double exponential function τ_f_ = 29 ± 3 ms (A_f_ = 84 ± 5%); τ_s_ = 280 ± 72 ms (*n* = 5) (Fig. 1b). These data indicate that the binding of either glutamate or glycine alone is not sufficient to allow a conformational state(s) of the receptor to which PA-S can bind and dissociate on a slow time scale.

Use-dependency is a characteristic feature of NMDAR channel blockers; however, the voltage-independent action of PA-S^10^,^13^ and voltage-dependent action of blockers indicate distinct sites of action at the NMDAR. To explore the PA-S interaction with the NMDAR channel, we analyzed the current following the termination of glutamate and steroid co-application (after-current) (Fig. 1c). This current is typical for voltage-dependent NMDAR inhibitors; however, in a broader sense it can be interpreted as a sudden release of an inhibitor from an activated receptor with the channel being either open or reaching the open conformation for a short duration of time before its closure^16^. Relative charge transferred during the after-current following 1 mM glutamate and PA-S (10, 30, 50, 100, 150, 300 μM) co-application was fitted by linear regression with a slope of −0.26 (Fig. 1d). The negative slope of the linear regression indicate “trapping” mechanism of action while the use-independent PA-S washout^13^ indicates opposing “foot in the door” mechanism (these terms characterize the molecular mode of interaction of two classes of voltage-dependent inhibitors with the ion channel^16^). This apparent contradiction may be explained by the fact that voltage-dependent ion channel inhibitors are assumed to block an open ion channel however steroids inhibit several conformational states associated with activated receptor (agonist activated with the ion channel open or closed^13^, and perhaps also the desensitized state of the receptor^17^).

### Effect of steroids on mutated receptor

We next analyzed the effect of PA-S on receptors phylogenetically closely related to the NMDAR. GluR0 is a potassium-selective prokaryotic glutamate receptor^18^. Glutamate-induced responses of this receptor exhibited profound desensitization that precluded a more detailed analysis; however, the superposition of the control responses and those made in the presence of PA-S indicate that this receptor was inhibited by the neurosteroid (Supplementary Fig. 1a). Similarly, glycine-activated GluN1-4a(F484A)/eGFP-GluN3A receptor^18^,^19^ was PA-S sensitive (IC_50_ = 27 ± 3 μM; *n* = 7), with a potency similar to that assessed for GluN1/GluN2B receptors (Supplementary Fig. 1b). GluN1-4a/GluN3A receptors exhibit rapid desensitization that hampers an analysis of PA-S inhibition. GluN1-4a(F484A) mutation reduces receptor desensitization and allows a more accurate analysis. Nevertheless, there was no significant difference in the degree of PA-S inhibition between the mutated and WT GluN1-4a subunit in combination with GluN3A. To further specify the site of inhibitory neurosteroid action at the NMDAR, we used truncated receptors. Truncation of the N- or C-terminal domain of the GluN1-1a and GluN2B subunits had no significant effect on the PA-S inhibition of NMDAR responses (GluN1/GluN2B: IC_50_ = 45 ± 1 μM (*n* = 20); GluN1ΔN/GluN2BΔN: IC_50_ = 41 ± 2 μM (*n* = 4) P = 0.174 (Supplementary Fig. 1c); GluN1ΔC/GluN2BΔC: IC_50_ = 34 ± 3 μM (*n* = 4) P = 0.9).

The results of our electrophysiological experiments on the GluN1/GluN2B receptors (Fig. 1) as well as on NMDARs with deleted N-terminal and C-terminal domains (Supplementary Fig. 1c) are compatible with a hypothesis that inhibitory steroids act at the transmembrane domain of the NMDAR. The following experiments aimed to identify amino acid residues of the NMDAR that are important for inhibitory steroid action. We have directed our attention to amino acids at the extracellular portion of the transmembrane domain because sulfated steroids do not readily cross the cytoplasmic membrane^12^ and have no obvious effect when intracellularly dialyzed^15^. Further, we have confined our attention to the first and the third transmembrane region (M) of the GluN1 and GluN2B subunits, as GluR0 receptors (which lack the M4^18^), are equally PA-S sensitive (Supplementary Fig. 1a).

**Figure 2:**
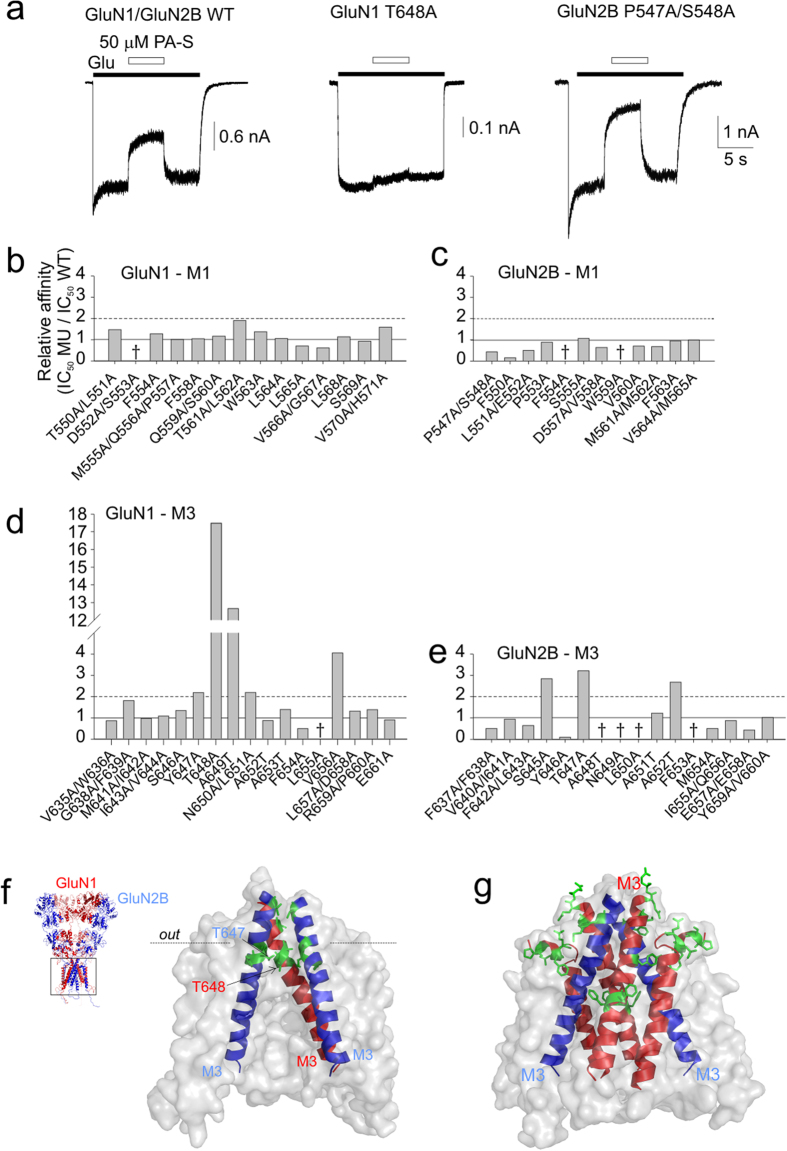
Mutations in the M1 and M3 regions of the GluN1 and GluN2B subunits alter the inhibitory potency of PA-S. (**a**) A representative response is shown for WT and mutated GluN1/GluN2B receptors. Responses mediated by GluN1(T648A)/GluN2B receptors were assessed as a difference between the current recorded in the presence of 1 mM Mg^2+^ (condition that abolished the spontaneous currents) and that recorded in the presence of 1 mM glutamate and 10 μM glycine. While the GluN1(T648A)/GluN2B receptors were only slightly affected by PA-S (50 μM) when compared to the WT receptor, the GluN1/GluN2B(P547A/S548A) receptors were more sensitive to the steroid. Graph of the relative PA-S affinity for NMDARs mutated in the M1 regions of GluN1 (**b**) and GluN2B (**c**) or mutated in the M3 regions of GluN1 (**d**) and GluN2B (**e**) are shown. Relative PA-S affinity was determined as the ratio of PA-S IC_50_ (Equation 1) determined for mutated receptors and the IC_50_ for the WT receptor (42 μM; *n* = 20). † indicates a non-responding mutation, i.e. a minimum of six GFP-positive cells responding to 1 mM glutamate application with currents <20 pA. (**f**) Amino acid residues whose mutations altered PA-S inhibition are depicted in a homology model of GluN1/GluN2B. For clarity, only the transmembrane region is shown. GluN1 is colored in light blue and GluN2B is colored in light red. Dashed line indicates the boundary between hydrophobic and hydrophilic regions of lipid as determined by the OPM database^63^. Residues whose mutation to alanine increased the IC_50_ for PA-S inhibition of GluN1/GluN2B receptor responses more than two–fold (to >84 μM) are highlighted in green. (**g**) Residues whose mutation to alanine decreased the IC_50_ for PA-S inhibition by half (to <21 μM) are highlighted in green.

Figure 2a shows that mutations in the membrane regions of the NMDAR affected the sensitivity to PA-S. Eight mutations (GluN1: Y647A, T648A, A649T, N650A/L651A, V656A; GluN2B: S645A, T647A, A652T) increased the PA-S IC_50_ values over two-fold relative to the WT receptor value (IC_50_ = 42 ± 1 μM (*n* = 20)). The greatest increase in the PA-S IC_50_ was observed for the mutation GluN1(T648A)/GluN2B, which reached 758 ± 27 μM (*n* = 8) (Fig. 2b–e). Amino acid residues whose mutation to alanine decreased PA-S affinity are located within or near the highly conserved SYTANLAAF motif^20^ forming the extracellular vestibule of the channel (Fig. 2f). HEK293 cells transfected with seven mutations (GluN1: L655A; GluN2B: F554A, W559A, A648T, N649A, L650A, F653A) either did not respond to glutamate application (1 mM) or the responses were too small to reliably assess the degree of PA-S inhibition (tested on a minimum of 6 cells). Eight mutations (GluN1: F654A; GluN2B: P547A/S548A, F550A, L551A/E552A, F637A/F638A, Y646A, M654A, E657A/E658A) decreased the PA-S IC_50_ values to less than half of the WT receptor value, with the mutation GluN2B(Y646A) increasing PA-S affinity to IC_50_ = 4.3 ± 0.7 μM (*n* = 10) (Fig. 2b–e). In addition, amino acid residues whose mutation to alanine increased PA-S affinity are located at the surface of the membrane regions (in contact with the membrane lipids), and 5 out of 8 are aromatic amino acids (Fig. 2g).

**Figure 3:**
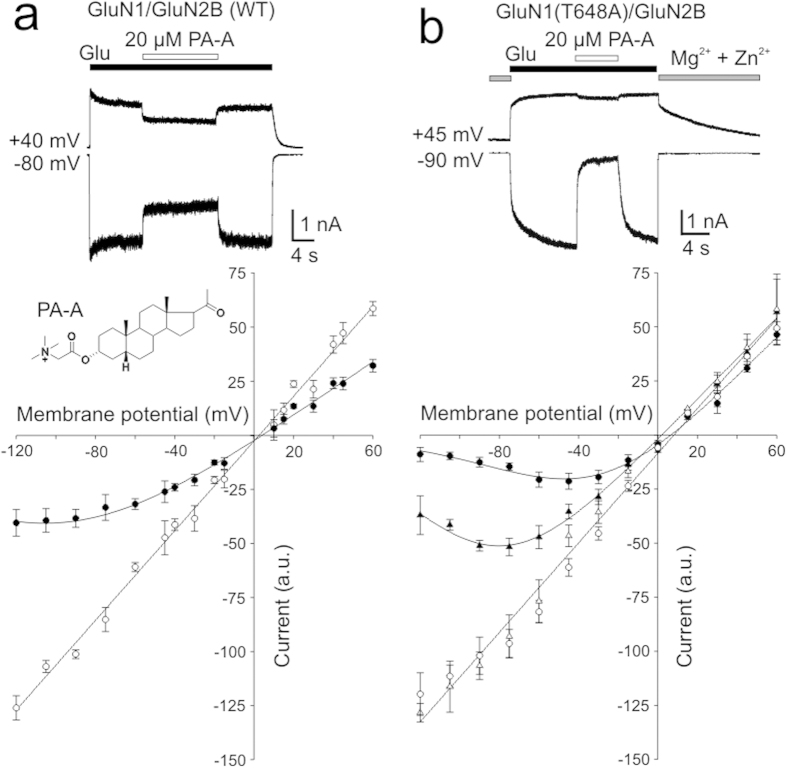
Current-to-voltage relation of the steroid action at the WT and mutated NMDARs. (**a**) Representative current of responses of GluN1/GluN2B receptors to glutamate (1 mM) recorded at –80 and +40 mV. Glutamate-evoked currents recorded at each of the membrane potentials indicated are reversibly inhibited by a co-application of PA-A (20 μM, indicated by the open bar). Plot of the amplitudes of control responses (open symbols) and those induced in the presence of PA-A (20 μM) (closed symbols) *versus* the holding potential. Control responses were fit by a linear equation and normalized to have a slope of 1. Responses recorded in the presence of PA-A were normalized with respect to their control and each cell fit to the Boltzmann equation (Eq. 2, 3, 4). Due to the weak voltage dependence of PA-A, the values of δ = 0.15 and PA-A K_d(0 mV)_ = 335 μM have to be interpreted cautiously. Data points are mean ± SEM for 6 cells. (**b**) GluN1(T648A)/GluN2B receptor responses induced by the washout of 1 mM Mg^2+^ and 30 μM Zn^2+^ (the condition blocking the agonist-independent NMDAR activity) and recorded in the presence of 1 mM glutamate and 10 μM glycine. Responses were reversibly inhibited by a co-application of PA-A (20 μM; indicated by the open bar) at a holding potential –90 mV. PA-A had only a small inhibitory effect at a holding potential +45 mV. Plot of the relative degree of PA-A inhibition *versus* the holding potential is shown for GluN1(T648A)/GluN2B (○) and GluN1(A649T)/GluN2B (Δ) receptor responses. Control responses were fit by a linear equation, responses recorded in the presence of PA-A were normalized with respect to the control response and fit to the Boltzmann equation (δ = 0.29, g_0_ = 0.83, and PA-A K_d(0 mV)_ = 340 μM for GluN1(T648A)/GluN2B receptor responses (*n* = 7); δ = 0.23, g_0_ = 0.93 and PA-A K_d(0 mV)_ = 745 μM GluN1(A649T)/GluN2B receptor responses (*n* = 8)). Data points are mean ± SEM.

The extracellular M3 regions of both the GluN1 and GluN2 subunits, within the SYTANLAAF motif, are considered to be a part of the external gate^21^. Because only the positively charged compounds can pass through the extracellular channel vestibule and bind inside the NMDAR channel pore in a voltage-dependent manner, we used in the next experiments 5β-pregnan-20-on 3α-yl-[(2-trimethylammonio)-ethanoate] (PA-A), a positively charged steroid with a ring structure identical to that of PA-S (Fig. 3a). Like PA-S, PA-A is a use-dependent NMDAR antagonist, showing an after-current following the termination of glutamate and PA-A co-application (data not shown). We have shown earlier that PA-A-induced inhibition is voltage independent from −60 to +60 mV^12^; however, at highly hyperpolarized membrane potentials (<−60 mV), the relative degree of PA-A inhibition increased (Fig. 3a). Fit to the Boltzmann equation (see Eq. 2, 3, 4) provided δ = 0.15, PA-A K_d(0 mV)_ = 335 μM, and g_0_ = 0.62 (*n* = 11). The weak voltage dependence of the inhibition of the WT NMDAR by a positively charged PA-A indicates that the binding site is located near to the outer border of transmembrane electric field. In contrast to the WT NMDAR, the effect of PA-A at GluN1(T648A)/GluN2B receptors was strongly voltage dependent ( δ = 0.29, K_d(0 mV)_ = 340 μM, g_0_ = 0.83; *n* = 7) (Fig. 3b). An intermediate voltage dependence was observed for GluN1(A649T)/GluN2B receptors (δ = 0.23, K_d(0 mV)_ = 745 μM, g_0_ = 0.93, *n* = 8) (Fig. 3b) and GluN1(V656A)/GluN2B receptors (δ = 0.19, K_d(0 mV)_ = 193 μM, g_0_ = 1.0 *n* = 8). In agreement with previous results, our data indicate that the ring of threonines GluN1(T648) and GluN2B(T647) (TTTT-ring) forms the narrowest portion of the ion channel^22^,^23^. Replacing threonine for a smaller residue of alanine makes the diameter of the open conformation of the channel wider so that the molecule of the steroid can move through the extracellular vestibule deeper into the channel pore. As expected for negatively charged PA-S, its effect was voltage independent for both the WT and GluN1(T648A)/GluN2B receptors (Supplementary Figure 2).

Mutations of amino acids in the SYTANLAAF motif of the AMPA receptor channels result in constitutive opening and cause neurodegeneration in the Lurcher mutant mouse^24^. A similar effect was described for mutations in this motif in the NMDAR^25^,^26^,^27^,^28^. An example of spontaneous activity recorded from HEK293 cells transfected with GluN1(T648A)/GluN2B receptors is shown in Supplementary Figure 3. In the absence of glutamate and glycine, 0.5–2 nA currents sensitive to Mg^2+^ appeared. These currents were only moderately sensitive to D-amino-5-phosphonopentanoic acid (D-AP5; 100 μM) and 7-CKA (100 μM), competitive antagonists at the glutamate and glycine binding sites of the NMDA^29^,^30^. Relative spontaneous activity (calculated as the ratio of current recorded in the absence of agonists to that recorded in the presence of 1 mM glutamate and 10 μM glycine) ranged from low levels to 87 ± 3% (*n* = 7), which was observed for GluN1(A649T)/GluN2B receptors. The degree of PA-S (200 μM) inhibition of spontaneous (13.8 ± 0.7%; *n* = 4) and agonist-evoked currents (13.6 ± 0.9%; *n* = 4) in HEK293 cells transfected with GluN1(T648A)/GluN2B receptors was not significantly different (P = 0.913). Of 71 functional mutations tested, 31 mutations exhibited relative spontaneous activity (RSA; >6%) (GluN1: T550A/L551A, F558A, W563A, V570A/H571A, I631A/L632A, S646A, Y647A, T648A^‡^, A649T^‡^, N650A/L651A, A652T^‡^, A653T^‡^, F654A, V656A^‡^, L657A/D658A^‡^, I664A/T665A, G666A/I667A; GluN2B: S539A/R540A, V545A/S546A, P553A^‡^, S555A^‡^, D557A/V558A, I630A/M631A, V632A/S633A, S645A^‡^, T647A^‡^, M654A, I655A/Q656A, E657A/E658A, Y659A/V660A^‡^, G665A/L666A; note that ^‡^ indicates the spontaneous activity described earlier)^25^,^26^,^27^,^28^,^31^. No correlation was found between the PA-S IC_50_ determined for the mutated receptors and the degree of spontaneous activity (Spearman correlation P = 0.113; r^2^ = 0.084) (Supplementary Fig. 3c). This suggests that the decrease of PA-S activity at the mutated receptors is not primarily due to the ability of these receptors to spontaneously open.

### Access of neurosteroids to the NMDAR

The amphipathic character of the PA-S molecule predicts this steroid’s propensity to form aggregates in aqueous solutions. Light scattering analysis of PA-S (5 nM to 150 μM) in ECS confirmed this prediction and indicated a presence of two particle sizes at >300 nM PA-S; since the longitudinal axis of the PA-S vdW surface is 1.56 nm and the steroid molecule is expected to be hydrated, we suppose that the small particle size (~2.5 nm) represents a single steroid molecule. Larger aggregates represent an agglomeration complex (Supplementary Fig. 4). Light scattering data indicate that the critical aggregate concentration (CAC) for PA-S is ~300 nM. By definition, at <CAC the monomer concentration of the steroid increases with the steroid added; however, at >CAC aggregates form and all additional steroid molecules added to the system go into aggregates^32^.

**Figure 4:**
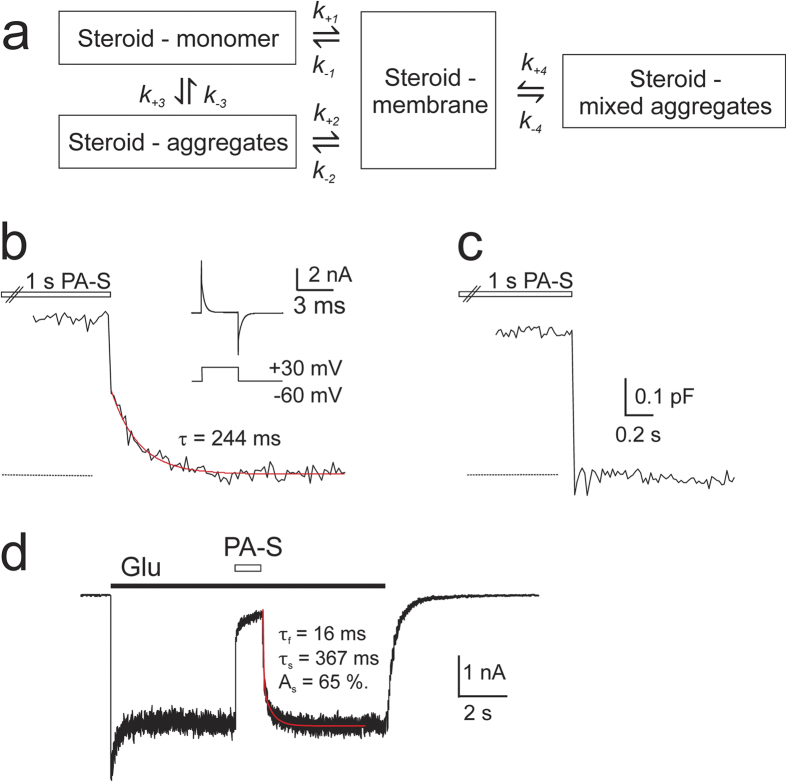
The rate of neurosteroid departitioning from the plasma membrane is fast. (**a**) Scheme of possible events taking place with steroid in the solution. The processes corresponding to the equilibria are: 1, monomer binding to the membrane; 2, aggregates binding to the membrane; 3, monomer-aggregates equilibrium; 4, membrane solubilization (steroid leaving the membrane carrying membrane components). *k* represents rate constants. Scheme adopted from^33^. (**b**) Currents induced by changes in the membrane holding potential from −60 to +30 mV for 3 ms at a frequency of 200 Hz were used to assess changes in the membrane capacitance induced by fast application of PA-S (150 μM) (inset). The slow component of the exponential fit to the offset of PA-S-induced capacitance change is shown for HEK293 cell attached to the surface of the cover glass (red line). Dashed line indicates cell capacitance before steroid application. (**c**) Changes in the membrane capacitance following fast application of PA-S in a lifted HEK293 cell. Dashed line indicates cell capacitance before steroid application. (**d**) Response induced in a lifted HEK293 cell transfected by GluN1/GluN2B receptors by fast application of glutamate (1 mM) was inhibited by PA-S (150 μM) with slow off kinetics after PA-S wash.

Our next experiments aimed to elucidate the mechanism that accounts for the apparent discrepancy between the PA-S concentration at which its monomer concentration changes with the added steroid (<CAC i.e ≤300 nM) and the PA-S dose-response relationship at NMDARs (10 to 300 μM). The system containing monomer and aggregate forms of the solute has the general properties of colloidal solutions; however, at sites located near the membrane, one must take into account local increases in the concentration of the monomer form of the steroid due to its diffusion from the cell membrane^33^ (Fig. 4a). This scheme assumes that after its association with the membrane, the steroid leaves the membrane as a monomer, aggregate and mixed aggregate. We used capacitance measurements to infer the rate of steroid association and exit from the membrane (Fig. 4b,c). Fast application of PA-S (150 μM) onto non-transfected HEK293 cells for 1 s resulted in ~3% capacitance increase during the steroid application; after the steroid washout, the capacitance returned to its original level with a double exponential time course (τ_f_ < 5 ms (likely affected by the rate of the solution exchange around the cell); τ_s_ = 222 ± 8 ms (A_s_ = 41 ± 2%); *n* = 7).

At PA-S concentrations relevant to its effect at the NMDAR, the vast majority of the steroid exists in rather large (~400 nm) aggregates (Supplementary Fig. 4). Consequently, it was expected that diffusion of a large molecular complex to and from the narrow space of the cell and dish contact would be slow and likely limit the rate of steroid-induced capacitance change. This assumption was confirmed when lifted cells (detached from the culture dish) were used (Fig. 4c). Under these conditions, only the fast component of the capacitance change after PA-S washout was resolved, and it remained too fast to be assessed accurately (τ < 5 ms; *n* = 5) (Fig. 4c).

Lifted GluN1/GluN2B transfected HEK293 cells were used to assess the rate of steroid association and dissociation from the receptor (Fig. 4d). In contrast to capacitance measurements on lifted cells (where only the fast component characterized the onset and offset of 150 μM PA-S induced capacitance changes), the onset of the response induced by 150 μM PA-S co-application with the glutamate was fitted by a double exponential function (τ_f_ = 10.8 ± 2.2 ms; τ_s_ = 480 ± 108 ms (A_s_ = 20 ± 4%); *n* = 5; significantly faster than in attached cells). The fast component of the onset of inhibition was significantly (P = 0.010) slower than the solution exchange time determined in lifted cells (see Methods) (Fig. 4d). Similarly, the off rate was best fitted by a double exponential function (τ_f_ = 14.3 ± 1.7 ms; τ_s_ = 311 ± 70 ms (A_s_ = 41 ± 6%); *n* = 5; significantly faster than in attached cells). These data indicate that the slow offset kinetics of PA-S inhibition are primarily due to the slow kinetics of steroid unbinding from the receptor and that steroid diffusion from narrow spaces (like the cell-dish contact) may further slow the macroscopic rate of steroid washout.

The equilibrium concentration of PA-S in the membrane was assessed biochemically using membrane ghosts prepared from rat erythrocytes. The aqueous PA-S concentration was reduced following equilibration with membrane ghosts (see Methods). The volume concentration of PA-S was calculated from the difference in the concentrations as described in the caption to Supplementary Fig. 5. Assuming that PA-S disperses only in the extracellular membrane leaf (see Methods), when [PA-S]_total_ = 55.7 μM (corresponding to the IC_50_ value) the membrane concentration [PA-S]_membrane_ = 4.32 nM/m^2^ (i.e., 2600 molecules/μm^2^). The flux J_out_ of steroid from the membrane into the aqueous phase is given by the product of [PA-S]_membrane_ and the rate k_off_ of steroid dissociation from the membrane. As the measured fast dissociation time constant τ_s_ was approximately 1 ms or faster, k_off_ ≥1000/sec and the flux J_out_ is at least 4.3 μM s^−1^ m^−2^.

At equilibrium, the dissociation of monomers from the membrane (flux J_out_) is balanced by two processes in the aqueous phase: the diffusion of monomers away from the membrane and aggregation of monomers into aggregates. This balance establishes a steady spatial profile of the concentration of PA-S monomer, with the highest concentration reached in the immediate vicinity of the membrane and the CAC (300 nM) reached far from the membrane. Estimates based on solving a simplified reaction-diffusion equation in the unstirred layer (Supplementary Text 1) indicate that the PA-S monomer concentration near the membrane exceeds 2 μM (see Supplementary Fig. 6). Depending on the value of k_off_ and on the rate of monomer aggregation, concentrations on the order of 10 μM may be reached. Lower estimates would be obtained if a significant portion of PA-S dissociated from the membrane in the form of aggregates.

## Discussion

**Figure 5:**
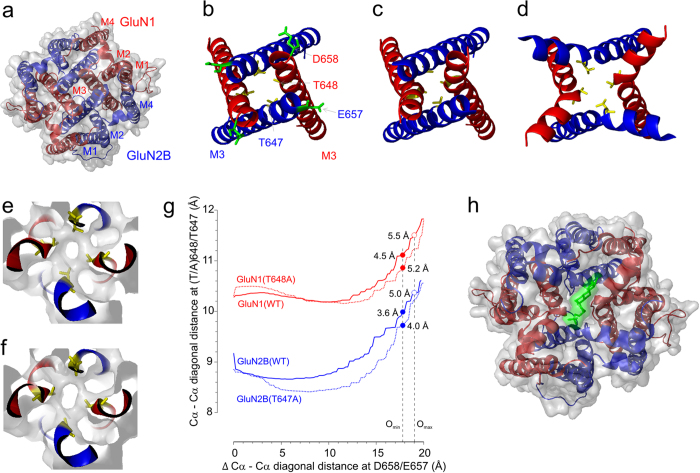
NMDAR channel opening and neurosteroid binding. (**a**) The membrane domains of the closed conformation of the NMDAR channel viewed from the extracellular side (GluN1 in red; GluN2B in blue). (**b**) The pore-forming segment (M3) of the GluN1/GluN2B receptor channel in a closed conformation. Residues T648 (GluN1) and T647 (GluN2B) at the narrow portion of the ion channel pore are shown as stick models (yellow) and residues D658 (GluN1) and E657 (GluN2B) at the most extracellular site of the M3 helices (green). (**c**) The position and shape of the M3 segments at the initial stage of opening induced by a stepwise (see Methods) increase in the transverse distance at the DEDE ring by a total of 1.0 Å. (**d**) The position and shape of the M3 segments at the open state induced by a stepwise increase in the transverse distance at the DEDE ring by a total 17.8 Å. (**e**) A slab of the molecular surface representation of the WT receptor at the region of the TTTT ring (as shown in d). (**f**) The representation of the open state of the GluN1(T648A)/GluN2B receptor induced by the same increase in the transverse distance as in the WT receptor (17.2 Å). (**g**) Comparison of the diagonal distances of the Cα atoms of T648 to T648 (GluN1) and T647 to T647 (GluN2B) of the WT receptor and A648 to A648 (GluN1) and T647 to T647 (GluN2B) of the mutated GluN1/GluN2B receptor induced by a stepwise increase in the diagonal distance between Cα atoms at the DEDE ring. The diameter of the channel pore represented by the channel vdW surface at the site of TTTT ring of the WT GluN1/GluN2B receptor (filled line) and GluN1(T648A)/GluN2B receptor (dashed line) calculated for the increase in the diagonal distance at D658 and E657 by 17.8 Å (O_min_; filled symbols) and 19.0 Å (O_max_; empty symbols). (**h**) Docking study of PA-S to the funnel formed by the M3 helices in the open channel configuration of the NMDAR. The most stable orientation is shown here.

Responses mediated by GluN1/GluN2B are inhibited by PA-A with signs of weak voltage dependence at highly hyperpolarized membrane potentials. In contrast, responses mediated by GluN1(T648A)/GluN2B receptors are blocked in a voltage-dependent manner (Fig. 3), indicating that the extracellular vestibule opens to a diameter that allows PA-A to move to the central pore cavity and block the ion flow. This difference was used to develop a model of the open state of the NMDAR channel and to infer on the steroid-NMDAR interaction. Firstly, we built a homology model of the NMDAR where T648 (GluN1) was mutated to alanine, and then 100 models differing in the diagonal distance (displacement up to 20 Å with 0.2 Å increment; see Methods) between the topmost M3 residues of both GluN1 and GluN2B (D658 to D658 (GluN1) and E657 to E657 (GluN2B); the DEDE ring) were obtained. The model with the vestibule open wide enough to allow PA-A to move from the extracellular site to the ion channel cavity was considered to mimic the open state of the NMDAR (Fig. 5a–e).

Our model almost certainly represents an oversimplification of the reality; it can nevertheless serve as a guide for deriving experimentally testable hypotheses. It predicts that even a small movement of the M3 helices from the closed to the open state first brings closer the A648 (or T648) residues of the GluN1 subunits, while having a relatively small effect on the displacement of the T647 residues of the GluN2B subunits (Fig. 5c,g). A further increase in the diagonal distance at the level of the DEDE ring (by ~10 Å) results in the bending of the M3 helices of both GluN1 and GluN2B, with only a small effect on the diagonal distance at the TTTT ring (Fig. 5d,g). Additional displacement at the level of the DEDE ring indicates that the narrowest portion of the extracellular vestibule of the GluN1(T648A)/GluN2B receptor that limits PA-A movement to the central channel cavity is at the site of the GluN1(T648A) and GluN2B(T647) ring (Fig. 5a,f). When the extracellular vestibule was sequentially opened by 17.8 Å to reach the diagonal distance 37 × 38 Å (C_α_ to C_α_) at the level of DEDE ring, the opening at the level of GluN1(T648A) and GluN2B(T647) was wide enough to allow the PA-A molecule to move from the extracellular vestibule to the central pore cavity. Taking into account the flexibility (rotation) of the AA residues, the minimal cross-section of this region was assessed to be 5.2 (GluN1 A-A) × 4.0 (GluN2B T-T) Å when vdW radii were taken into account and 10.9 × 10.0 Å when C_α_ to C_α_ was measured (Fig. 5f,g; O_min_). The model for the WT GluN1/GluN2B receptor for the same displacement at the DEDE ring, as identified for the GluN1(T648A)/GluN2B receptor (17.8 Å), predicts the narrowest extracellular vestibule dimension to be 4.5 × 3.6 Å when vdW radii are taken into account and 11.1 × 9.7 Å when C_α_ to C_α_ is measured (Fig. 5d,e,g; O_min_). This is the smallest theoretical opening, since the displacement was selected in a way that allowed the forces between the steroid and the protein structure of the channel to be non-repulsive. The asymmetric position of the GluN1(T648) and GluN2B(T647) in the open conformation of the NMDAR ion channel explains the different contribution of GluN1 and GluN2B to the receptor inhibition by PA-S.

Estimates of the narrowest dimension of the extracellular vestibule of the WT NMDAR based on a model of the mutated receptor is biased because it is quite likely that the opening of the mutated receptor is actually wider than would just suffice to allow PA-A to move across the vestibule. An alternative approach to estimating the dimension of the vestibule was based on an assumption that the open state of the WT GluN1/GluN2B receptor is just sufficient to allow the molecule of PA-A to move across the vestibule. Using the same approach as above, a stepwise increase in the diagonal distance at the level of the DEDE ring of the WT receptor by 19 Å (to reach the diagonal distance 38 × 39 Å (C_α_ to C_α_)) induced an opening at the level of the TTTT ring sufficient to allow the steroid molecule to move from the extracellular vestibule to the pore central cavity. The upper limit of the narrowest extracellular vestibule dimension was estimated to be 5.5 × 5.0 Å when vdW radii were taken into account and 11.5 × 10.2 Å when C_α_ to C_α_ was measured (Fig. 5g; O_max_).

Our proposed model of the WT NMDAR channel is compatible with previous estimates of the minimum cross-section of the channel permeation pathway in the conducting states 6.0 Å^34^, 5.5 Å^35^, and ~4.5 × 5.7 Å^36^ and within the limits proposed for the channel diameter at the level of the activated extracellular vestibule, which was estimated to be 7.3 Å^35^ and 11 Å^16^. Recently, molecular dynamic (MD) simulations were used to simulate the open conformation of the NMDAR channel^37^. In agreement with our model, the results of the MD simulations suggest a wide open extracellular vestibule of the pore; however, the dimensions of the narrowest region of the MD model (2 × 2 Å obtained by HOLE program–which should be equivalent to vdW and comparable to about 8 × 8 Å between C_α_ atoms) indicate that the NMDAR channel has not reached the open state yet.

Spontaneous activity was described earlier for mutations within or in close proximity to the SYTANLAAF motif 25,26,27,28,31. In agreement with the experimental data, our model of the NMDAR indicates that the TTTT region is an important site for stabilizing the closed conformation of the ion channel. At the initial stages of channel opening, the original square geometry of the region changes first to a more compact diamond shape with a smaller pore diameter as the upper parts of the inner helices bend (Fig. 5c,d,g). The compact geometry allows an optimal distance to form vdW contacts as well as hydrogen bonds among the side-chain atoms of threonine residues, and as the distance between the helices grows further, nonsymmetric opening/gating at these threonine residues progresses. These data agree well with the results of experiments in which the cysteine accessibility method and helix cross-linking were used to show the asymmetric contribution of the M3 segments from the GluN1 and GluN2 subunits to the opening of the extracellular NMDAR channel vestibule^26^,^38^. In contrast to the WT results, in mutants, the non-symmetric behavior and the reduction of pore diameter in the gating region during the initial steps of opening are less prominent (Fig. 5g). The asymmetric model of channel opening provides the structural basis for the experimentally observed functional and pharmacological differences between the GluN1 and GluN2B subunits the spontaneous activity differed 3.7-fold between GluN1(T648A)/GluN2B and GluN1/GluN2B(T647A) receptors, and the value of the PA-S IC_50_ differed 5.4-fold.

Pregnanolone metabolites (allopregnanolone, pregnanolone, epipregnanolone, etiocholanone) sulfated in the C3-position are found in plasma in the nM range with small gender differences^39^,^40^. These compounds are also synthetized in the nervous tissue^41^,^42^ and are thought to act locally to modulate neuronal activity^5^. Sulfated pregnanolone metabolites were shown to have inhibitory action on GABA receptors and ionotropic glutamate receptors^8^,^43^. The relatively low potency of these compounds at the NMDAR may be misleading, since its aggregate concentration (representing the majority of the steroid in the water solution at biologically relevant concentrations) is likely to be much higher than that of its monomeric form (relevant for drug-receptor interaction). The calculations based on experimental data indicate that at the PA-S concentration corresponding to its IC_50_ at NMDARs, 99.5% of molecules present in the ECS will be in aggregates. However, at the membrane surface, the monomeric form is expected to be increased to the μM range (see Supplementary Fig. 6). The results of the docking of PA-S to the site within the funnel formed by pore-forming helices indicate that this is indeed the case (Fig. 5h).

The docking did not suggest a single ligand binding site within the funnel, indicating that the appropriate size of the steroid molecule with respect to the channel vestibule is more critical than interactions of the steroid molecule with specific amino acids at this site. This agrees well with structural experiments indicating that the charge of the pregnanolone derivatives, as well as the substitution of the sulfate at C3 and H at C5, C7, C11, C17 by different residues, had only a small effect on the steroid potency to inhibit NMDAR responses^12^,^44^,^45^,^46^.

The results of our electrophysiological experiments are compatible with steroid binding in the ion channel pathway at the receptor extracellular vestibule (SYTANLAAF motif). This is a highly conserved region among ionotropic glutamate receptors, and in the antagonist-bound state it occludes the ion channel permeation pathway^21^,^27^,^47^. In AMPA receptors, a mutation in this region (Lurcher mouse) induces neurological symptoms characterized on the molecular level by spontaneously opening ion channels^24^,^48^. Similarly, this region is a critical determinant of channel gating in NMDARs^27^. Even though the site of action of neurosteroids (the extracellular vestibule) is distinct from that described for some voltage-dependent blockers such as Mg^2+^ 49,50, their mechanism of action is similar to that of blockers; i.e., the site is accessible upon receptor activation (use-dependent effect) and the agonist cannot dissociate with the steroid bound^51^.

Synthetic homologues of naturally occurring pregnanolone sulfate inhibit NMDAR responses, cross the blood brain barrier, protect neurons against acute NMDA-induced cell death, improve behavioral performance in cognitive tasks, and, in addition, exhibit sedative, anticonvulsant, and analgesic properties^9^,^44^. In contrast to voltage-dependent inhibitors, neurosteroids do not induce psychomimetic-like behavior when administered to control experimental animals and, surprisingly, they ameliorate psychomimetic behavior induced by systemic MK-801 administration^11^. This study shows that: a) inhibitory neurosteroids bind at the extracellular channel vestibule of the NMDAR, b) allosteric inhibition is dependent on the receptor activation, and c) the inhibition involves stabilization of the NMDAR channel structure in an open blocked conformation. The mechanism of action presented here provides a molecular blueprint for improving the design of therapeutic compounds targeting the NMDAR.

## Materials and methods

### Transfection and maintenance of cells

Human embryonic kidney (HEK293) cells were transfected with expression vectors containing: GluN1–1a (GenBank accession number U08261)^52^; GluN2B (GenBank accession number M91562); GluN1-4a(F484A), (generous gift from Dr. J. J. Woodward)^53^ (Smothers & Woodward, 2009); GluN3A (generous gift from Dr. S. A. Lipton)^54^ (GenBank accession number NM_138546); GluN1ΔN with deleted 354 amino acids in the NTD (32 to 386); GluN1ΔC with an introduced stop codon in 838 of the CTD GluN2BΔN) with deleted 354 amino acids in the NTD (32 to 386); GluN2BΔC with an introduced stop codon in 844 of the CTD^55^; GluR0 (GenBank accession number *Synechocystis* PCC 6803) (generous gift from Dr. M. L. Mayer)^18^; and green fluorescent protein (GFP) (pQBI 25, Takara, Tokyo, Japan) genes, as described previously^12^. The Quick-Change site-directed mutagenesis kit (Agilent Technologies, Santa Clara, CA, USA) was used to generate specific point mutations in the M1/M3 region according to the manufacturer’s instructions using manually designed primers purchased from Sigma. Modified DNA plasmids were transformed into competent XL10-Gold *E.coli* cells, positive clones were selected, and isolated DNA was sequenced. Transfected cells were revealed by GFP epifluorescence. All mutations were verified by DNA sequencing (Macrogen, Seoul, Korea or SeQme, Dobris, Czech Republic). The amino acids are numbered according to the full-length protein, including the signal peptide, with the initiating methionine as 1.

### Electrophysiological recording

Experiments were performed 24–48 hrs after transfection on cells transfected with GluN1/GluN2B/GFP plasmids. Whole-cell voltage-clamp recordings from HEK293 cells were made with a patch-clamp amplifier (Axopatch 200B; Axon Instruments, Inc., Foster City, CA) after a capacitance and series resistance (<10 MΩ) compensation of 80–90%.

Patch pipettes (3–5 MΩ) were filled with a solution containing (in mM): 120 gluconic acid, 15 CsCl, 10 BAPTA, 10 HEPES, 3 MgCl_2_, 1 CaCl_2_, and 2 ATP-Mg salt (pH-adjusted to 7.2 with CsOH). When GluR0 receptor responses were studied, intracellular solution contained (in mM): 120 gluconic acid, 15 KCl, 10 BAPTA, 10 HEPES, 3 MgCl_2_, 1 CaCl_2_, and 2 ATP-Mg salt (pH-adjusted to 7.2 with KOH). Extracellular solution (ECS) contained the following (in mM): 160 NaCl, 2.5 KCl, 10 HEPES, 10 glucose, 0.2 EDTA, and 0.7 CaCl_2_ (pH-adjusted to 7.3 with NaOH). Glycine (10 μM), an NMDAR co-agonist, was present in the control and test solutions. All (PA-S and PA-A) solutions were made from freshly prepared 20 mM PA-S and 5 mM PA-A stock in dimethyl sulfoxide (DMSO). The final dilution to ECS was made at 50 °C and followed by 1 min sonication (SONOREX DIGITEC DT 100/H, BADELIN electronic, Berlin, Germany). The same concentration of DMSO was added to all extracellular solutions. Drug applications were made with a microprocessor-controlled multibarrel fast-perfusion system. Changes in the glutamate-induced current in HEK293 cells transfected by GluN1/GluN2B subunits were used to estimate the rate of solution exchange around the cell. Standard ECS was switched to the ECS diluted by water (50%) during glutamate application. The osmolarity was adjusted by sucrose to match the standard ECS. Glutamate (1 mM), glycine (10 μM) and pH was maintained constant in both solutions. The solution exchange rate was estimated to be τ = 4.3 ± 0.3 ms (*n* = 6) for lifted cells and τ = 12.0 ± 0.9 ms (*n* = 6) for the attached cells.

Experiments were performed at room temperature (21–25 °C). Data were collected (low-pass filtered at 2 kHz and sampled at 10 kHz) and analyzed using pClamp 10 (Molecular Devices). Unless otherwise mentioned, error bars represent SEM; statistical comparison of groups was performed using Student’s t-test (p < 0.05 was used to determine the significance).

The IC_50_ was determined from a single dose of a steroid using the formula
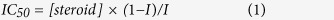
where I is the relative degree of inhibition and *[steroid]* is the steroid concentration used. Calculations of IC_50_ were made assuming 100% inhibition at saturating steroid concentration this is valid for both PA-S and PA-A^12^,^13^.

The *I–V* relation of the PA-A inhibitory effect was fitted to the Boltzmann function equation of the form:
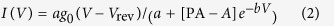
in which *g*_*0*_ is the estimated conductance of the NMDAR whole-cell response in the absence of extracellular PA-A, *V*_*rev*_ is the reversal potential of NMDA-induced current and *a*, *b* are parameters with the following interpretation:
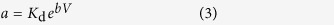
where *K*_d_ represents the apparent dissociation constant for PA-A binding to the NMDAR at a given membrane potential (*V*) and:
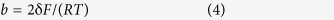
where δ indicates the apparent electrical distance of the PA-A binding site from the outside of the membrane and *F*, *R* and *T* have their standard thermodynamic meanings^56^. Unless otherwise noted, recordings were performed at a holding potential of −60 mV (values of the holding potential were corrected for the liquid junction potential, 14 mV).

Membrane capacitance was estimated by examining the integrated capacitive current (sampled at 30 kHz at 10 kHz filter cutoff frequency) evoked by depolarizing or hyperpolarizing voltage pulses from −60  mV to +30 mV for 3 ms delivered at 200 Hz.

All drugs, unless otherwise stated, were purchased from Sigma-Aldrich (St. Louis, MO, USA). PA-S and PA-A were synthesized as described previously^12^,^57^. The structures of all synthesized steroids were confirmed by IR, NMR, MS and HR-MS spectra.

### Light scattering analysis

Light scattering (Zetasizer Nano ZS, Malvern Instruments, United Kingdom) was used to characterize particle size of PA-S in ECS. PA-S was dissolved in DMSO at a concentration of 20 mM and then in the ECS at a final concentration of 5 nM to 150 μM PA-S. This solution was sonicated (Ultrasonic Sonorex Super RK 103 H, Bandelin electronic GmbH & Co. KG, Germany) for 1 min. Light scattering analysis was repeated six times for each sample at 25 °C.

### Analysis of the steroid concentration in the cytoplasmic membrane

All animal manipulations were conducted in accordance with the Animal Protection Code of the Czech Republic and corresponding directives of the European Community Council (2010/63/EC). Experiments were approved by the Animal Care and Use Committee of the Institute of Physiology of the Academy of Sciences of the Czech Republic. Blood was collected from one adult Wistar rat, heparinized, and spun down at 1,000 g for 10 min. Cells were washed once with PBS, briefly spun down and the pellet resuspended in a minimum amount of ECS. Cells were counted and 200 μl aliquots (~3*10^9^ cells) were taken for the experiment. Cell membranes were prepared by 30 min erythrocyte lysis in distilled water followed by centrifugation at 25,000 g for 10 min and resuspension in 200 μl of ECS. Cell membranes were incubated for 2 min with PA-S solution in ECS at the final volume of 500 μl, followed by a spin down at 20,000 g for 2 min. Supernatants were taken for liquid chromatography-mass spectrometry analysis (HPLC-MS). Solution of PA-S without membranes served as a control. HPLC-MS analysis was performed on a system consisting of Accela 1250 pump, Open Accela autosampler and Orbitrap Velos mass spectrometer (Thermo Scientific, USA). The analytes were separated on the Kinetex C18 column 100 mm × 2.1 mm × 1.7  μm and the mobile phase (solvent A: aqueous solution of acetic acid (0.1%); solvent B: methanol) eluted in isocratic solution (ratio 90:10) at a flow rate of 150 μl/min. The column temperature was maintained at 25 °C. The injection volume was 10 μl. The mass spectrometer equipped with an electrospray ion source was used for the detection of analytes in the selective reaction monitoring (SRM) mode in negative ionization. The parameters of the mass spectrometer were optimized as follows: spray voltage −2500 V (negative ionization mode), temperature of ion transfer tube 350 °C, temperature of H-ESI vaporizer 350 °C, pressure of sheath gas (nitrogen) 40 psi, flow of auxiliary gas (nitrogen) 15 arbitrary units. The data were acquired and processed using the software Xcalibur 2.1.0 (Thermo Scientific, USA).

### Molecular modeling of steroids

3-D structures of steroids were determined using Hyperchem 8 (Hypercube, Gainesville, FL, USA). The molecular geometry and partial charges were optimized by a semiempirical method PM3 considering the appropriate total charge of a molecule and singlet state. Subsequently, a general protocol for obtaining the lowest energy conformers by simulated annealing was used: optimized starting structure was subjected to a dynamic run (0.2 ps heating from 300 to 1000 K), 1 ps equilibration, and 2 ps cooling to 200 K, followed by energy minimization. Every subsequent run started from the previously minimized structure. A set of 100 structures was obtained for each compound, from which the lowest energy conformation was taken. The Polak-Ribiere conjugate gradient method for energy minimizations was used for convergence (less than 0.04 kJ. M^−1^ RMS force). Force field MM+ was used for all computations. All calculations were done *in vacuo*.

### Homology modeling

For the homology model of the NMDAR the crystal structure of AMPA and NMDARs were used^21^,^58^,^59^. The missing residues were added using the MODELLER 9v6 suite of programs^60^, resulting in a full length 3D structure of the receptor. The AMPA domain (GluA2) amino acid sequence was aligned with the GluN1 and GluN2B sequences using clustalw2^61^ program, and this alignment and the NMDA crystal structure were used to prepare the full length NMDAR models (3310 residues in total).

### Modeling of ion channel open state

Molecular modeling features of MODELLER package, including the conjugate gradient optimization and molecular dynamics, were used for a study of pore opening. Opening of the channel was modeled by elongating the distance between pairs of GluN1 and GluN2B domains separately (anchored by the Cα atoms of the topmost residues within the pore-forming helices) as well as the concerted opening involving both pairs. A stepwise optimization of the structure was employed; the exact protocol consisted of a series of conjugate gradient optimizations, short MD, followed by a conjugate gradient optimization for each increment of the above mentioned distance. The distance was varied within −5 Å to 20 Å from the original closed conformation of the NMDAR homology model. We decided to include secondary structure restraints for the innermost helices forming the pore. Restraining the entire inner helices presents an “extreme case” that makes the central helices more rigid in full length by restraining their alpha helical secondary structure. The most effective attempt restrained only the top part of the helices: the secondary structure was restrained for the topmost 8 residues of both GluN1 and GluN2B pore-forming helices and the distance changed in 0.2 Å increments. These simple restraints (only one/two interatomic distances and the secondary structure) were used in order to introduce as small a perturbation to the mechanism of channel opening as possible. The relative direction of the movement of pore-forming helices or the interdomain orientation was thus not restrained.

### Ligand docking

The docking of the PA-S and PA-A steroids to a series (intermediates of the opening models) of NMDAR homology structures was performed using the autodock suite of programs. The ligand atomic charges were obtained systematically by a standard quantum chemical procedure by HF/6-31G* electrostatic potential fit at the B3LYP/6-31G* gradient optimized geometry. Steroids were docked into the homology model of GluN1/GluN2B receptor using Python Molecular Viewer (PMV 1.5.6 rc3)^62^. Each steroid was allowed to sample docking poses in a box (70 × 70 × 90 grid points with 0.375 Å spacing) centered at the level of the gating residues. The residues at the surface of the forming channel were considered flexible. Four genetic algorithm docking runs of 50 were performed and data combined to find the most probable ligand “binding site”. In order to accurately identify the minimum necessary displacement corresponding to an open state, a local search was performed with ligand positioned between the gating residues.

## Additional Information

**How to cite this article**: Vyklicky, V. *et al.* Block of NMDA receptor channels by endogenous neurosteroids: implications for the agonist-induced conformational states of the channel vestibule. *Sci. Rep.* **5**, 10935; doi: 10.1038/srep10935 (2015).

## Supplementary Material

Supplementary Information
