# Supplementary material for: Block of NMDA receptor channels by endogenous neurosteroids: implications for the agonist induced conformational states of the channel vestibule
Source: Sci Rep. 2015 Jun 18;5:10935. doi: 10.1038/srep10935 (PMC4471902; doi:10.1038/srep10935)
Supplement: Supplementary Information [file srep10935-s1.pdf]

Title:

Block of NMDA receptor channels by endogenous neurosteroids: implications for the agonist-induced conformational states of the channel vestibule.

Author listing:

Vojtech Vyklicky[1], Barbora Krausova[1], Jiri Cerny[1], Ales Balik[1], Martin Zapotocky[1], Marian Novotny[2], Katarina Lichnerova[1], Tereza Smejkalova[1], Martina Kaniakova[1], Miloslav Korinek[1], Milos Petrovic[1,5,6], Petr Kacer[3], Martin Horak[1], Hana Chodounska[4], and Ladislav Vyklicky[1]

[1] Institute of Physiology, AS CR, Videnska 1083, 142 20 Prague 4, Czech Republic
[2] Charles University in Prague, Faculty of Science, Albertov 6, 128 43 Prague 2, Czech Republic
[3] Institute of Chemical Technology – Prague, Technicka 5, 166 28 Prague, Czech Republic
[4] Institute of Organic Chemistry and Biochemistry, AS CR, Flemingovo nam. 2, 166 10 Prague 2, Czech Republic
[5] School of Pharmacy and Biomedical Sciences, University of Central Lancashire, Preston, PR1 2HE, UK
[6] Institute of Medical Physiology, School of Medicine, University of Belgrade, Visegradska 26/II, 11000 Beograd, Srbija

Corresponding author: Ladislav Vyklicky, M.D., Ph.D., DSc.,
Institute of Physiology, AS CR
Videnska 1083, 142 20 Prague 4, Czech Republic
Phone: (+420) 24106 2450
Email: vyklicky@biomed.cas.cz

Supplementary Information

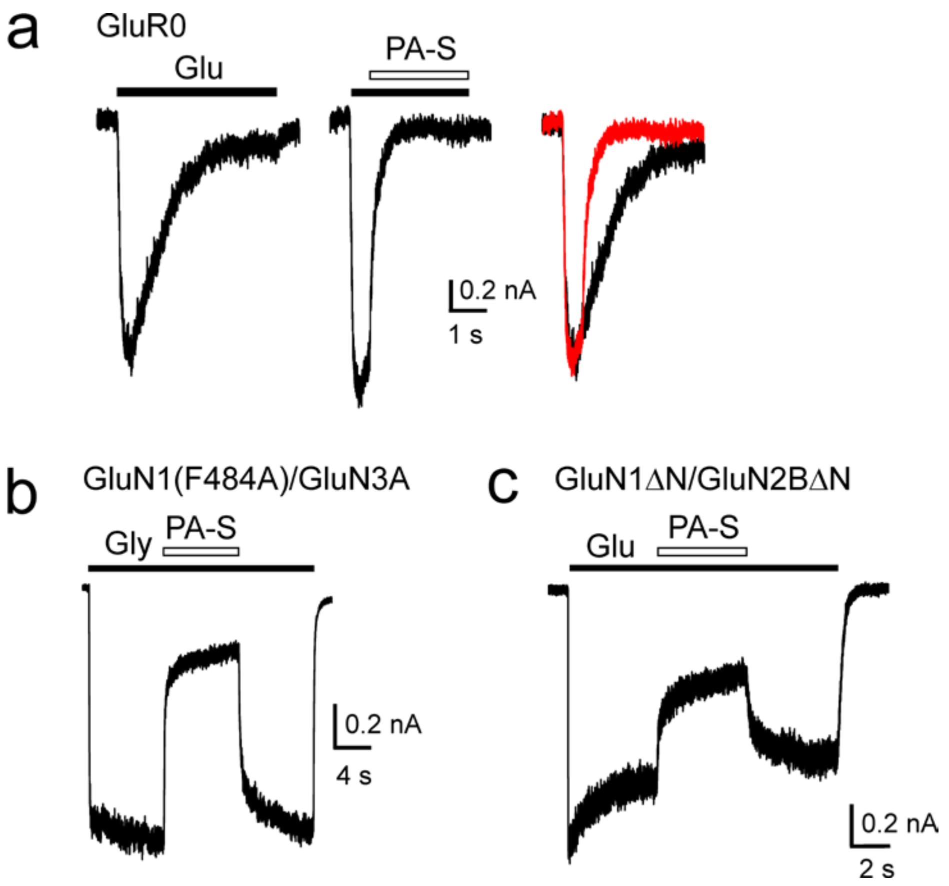

Supplementary Figure 1 | Sensitivity of ancestral and truncated NMDARs to PA-S. (**a**) Representative current responses of GluR0 receptor (glutamate-activated K^+ selective channel) to 1 mM glutamate (left) and to glutamate made in the presence of PA-S (300 µM) (middle). Control response to glutamate (black) and to glutamate made in the presence PA-S (red) are shown overlaid (right). (**b**) Representative current response of GluN1-4a(F484A)/GluN3A (glycine-activated glutamate receptor) to 100 µM glycine, its co-application with PA-S (50 µM), and steroid wash-out. (**c**) Representative current response of GluN1-1a/GluN2B receptor subunits with deleted N-terminal to 1 mM glutamate, its co-application with PA-S (50 µM), and steroid wash-out.

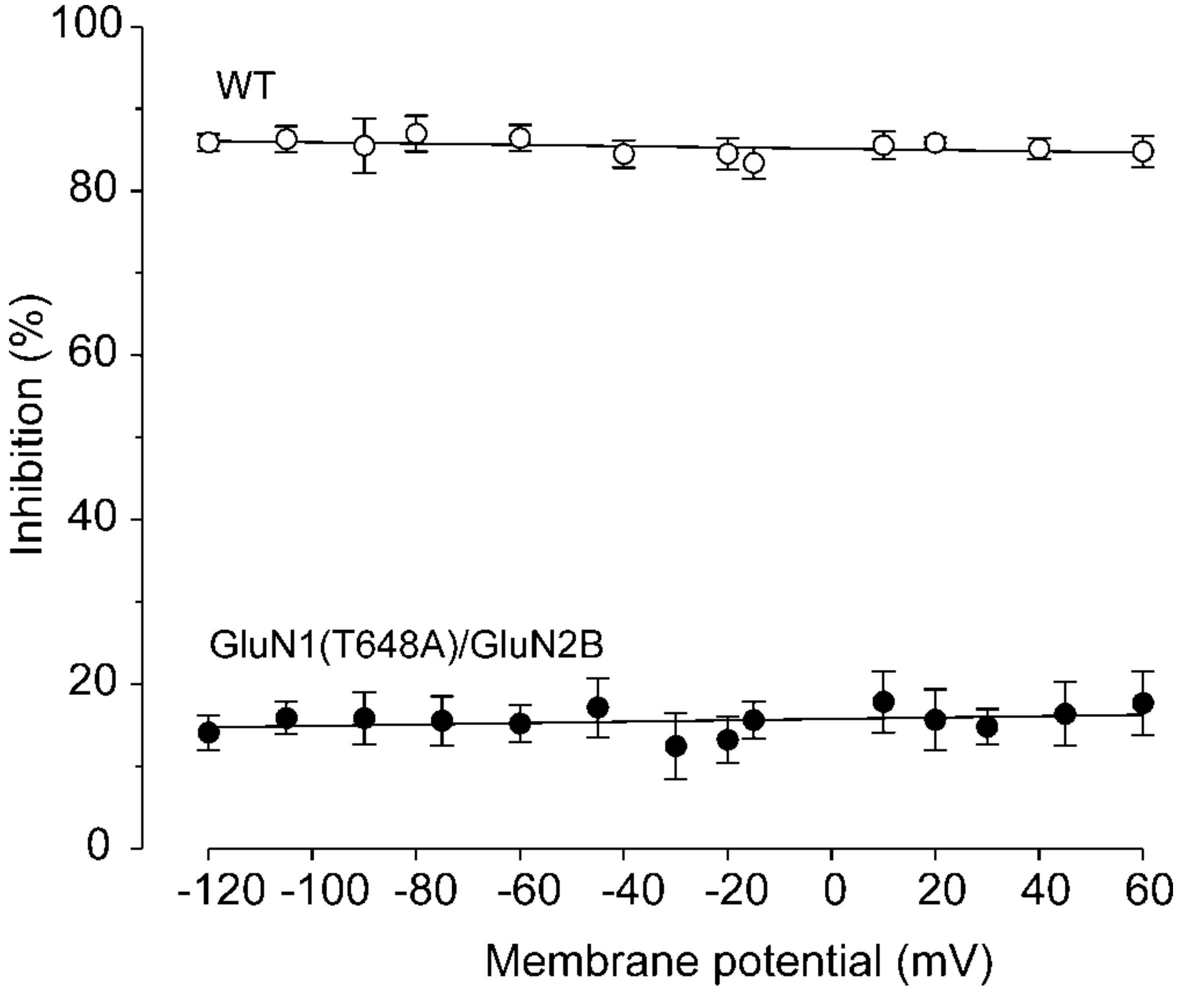

Supplementary Figure 2 | PA-S is a voltage-independent inhibitor of NMDA receptors. Plot of the mean PA-S (200 µM) inhibition of responses induced in GluN1/GluN2B (WT) and GluN1(T648A)/GluN2B receptors by 1 mM glutamate *versus* holding potential. Data points were fitted to the linear equation of the following form: $y = 85.2 - 7.9 * 10^{-3} * x$ (WT) and $y = 15.8 + 8.5 * 10^{-3} * x$ (GluN1(T648A)/GluN2B receptors).

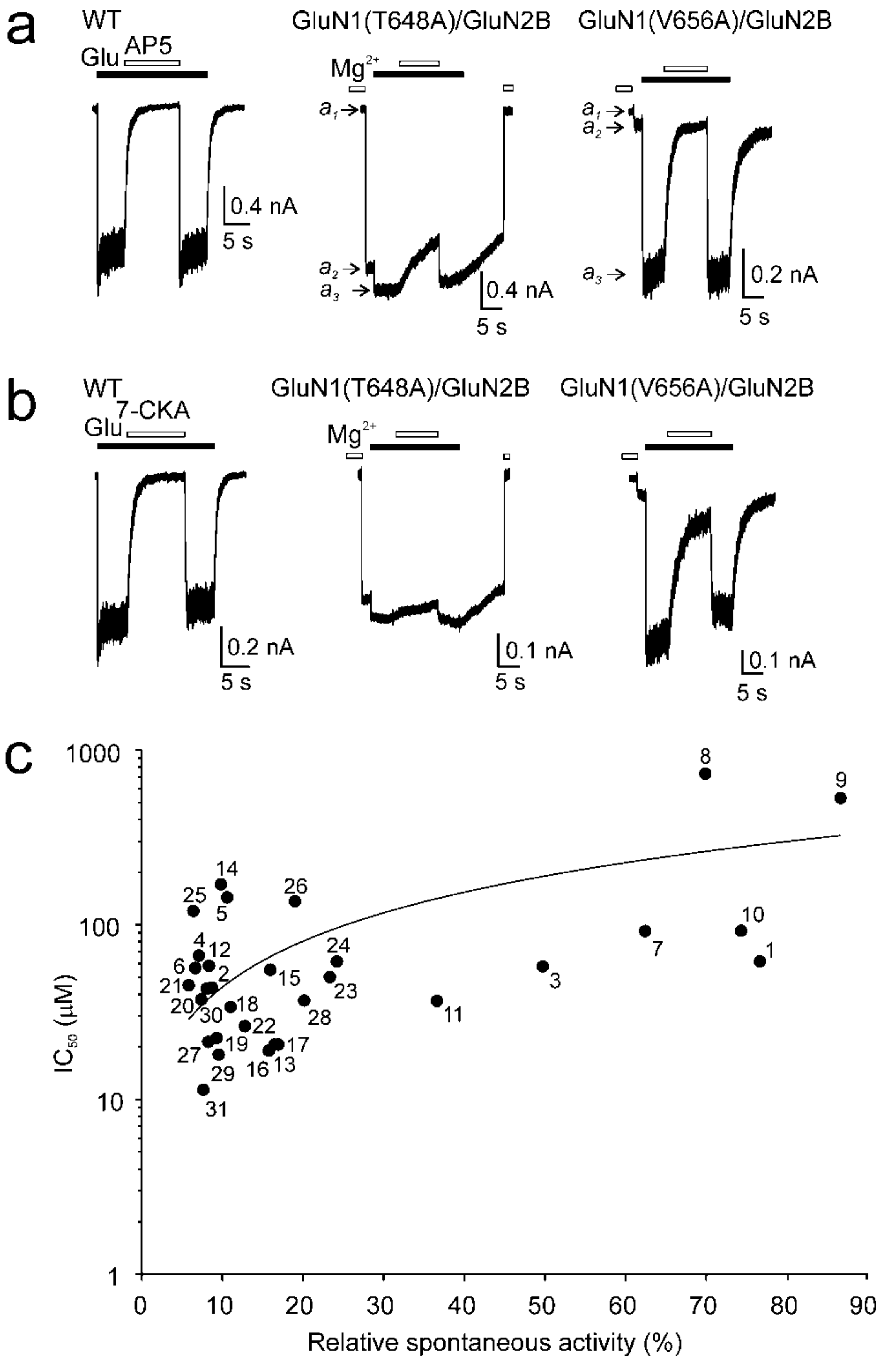
a
WT
Glu
AP5
0.4 nA
5 s
GluN1(T648A)/GluN2B
Mg2+
a1
a2
a3
0.4 nA
5 s
GluN1(V656A)/GluN2B
a1
a2
a3
0.2 nA
5 s
b
WT
Glu
7-CKA
0.2 nA
5 s
GluN1(T648A)/GluN2B
Mg2+
0.1 nA
5 s
GluN1(V656A)/GluN2B
0.1 nA
5 s
c
IC50 (μM)
Relative spontaneous activity (%)

Supplementary Figure 3 | Pharmacological properties of mutated NMDARs exhibiting decreased sensitivity to PA-S. (**a**) Representative current responses of GluN1/GluN2B (WT), GluN1(T648A)/GluN2B and GluN1(V656A)/GluN2B receptors to glutamate (1 mM) and glutamate with D-AP5 (100 µM) (duration of inhibitor and glutamate application is indicated by filled and open bars respectively). (**b**) The effect of 7-CKA (100 µM) applied simultaneously with 1 mM glutamate on responses of GluN1/GluN2B (WT), GluN1(T648A)/GluN2B and GluN1(V656A)/GluN2B receptors. GluN1(T648A)/GluN2B and GluN1(V656A)/GluN2B receptor responses were assessed as the difference between the current recorded in the presence of 1 mM Mg^{2+} and current induced in the presence of 1 mM glutamate and 10 µM glycine. (**c**) Plot of the PA-S IC_{50} determined for the mutated receptors as a function of the degree of relative spontaneous activity (RSA). RSA was calculated according to the following formula RSA = $(a_2 - a_1) / (a_3 - a_1)$ where $a_1, a_2,$ and a_3 are currents recorded in the presence of 1 mM Mg^{2+}, in the absence of added Mg^{2+}, and 1 mM glutamate, respectively. The following mutations exhibited RSA >6% GluN1: 1 (T550A/L551A), 2 (F558A), 3 (W563A), 4 (V570A/H571A), 5 (I631A/L632A), 6 (S646A), 7 (Y647A), 8 (T648A), 9 (A649T), 10 (N650A/L651A), 11 (A652T), 12 (A653T), 13 (F654A), 14 (V656A), 15 (L657A/D658A), 16 (I664A/T665A), 17 (G666A/I667A). GluN2B: 18 (S539A/R540A), 19 (V545A/S546A), 20 (P553A), 21 (S555A), 22 (D557A/V558A), 23 (I630A/M631A), 24 (V632A/S633A), 25 (S645A), 26 (T647A), 27 (M654A), 28 (I655A/Q656A), 29 (E657A/E658A), 30 (Y659A/V660A), 31 (G665A/L666A). Data were fitted by linear regression. There is no significant relationship between IC_{50} determined for the mutated receptors and the degree of spontaneous activity.

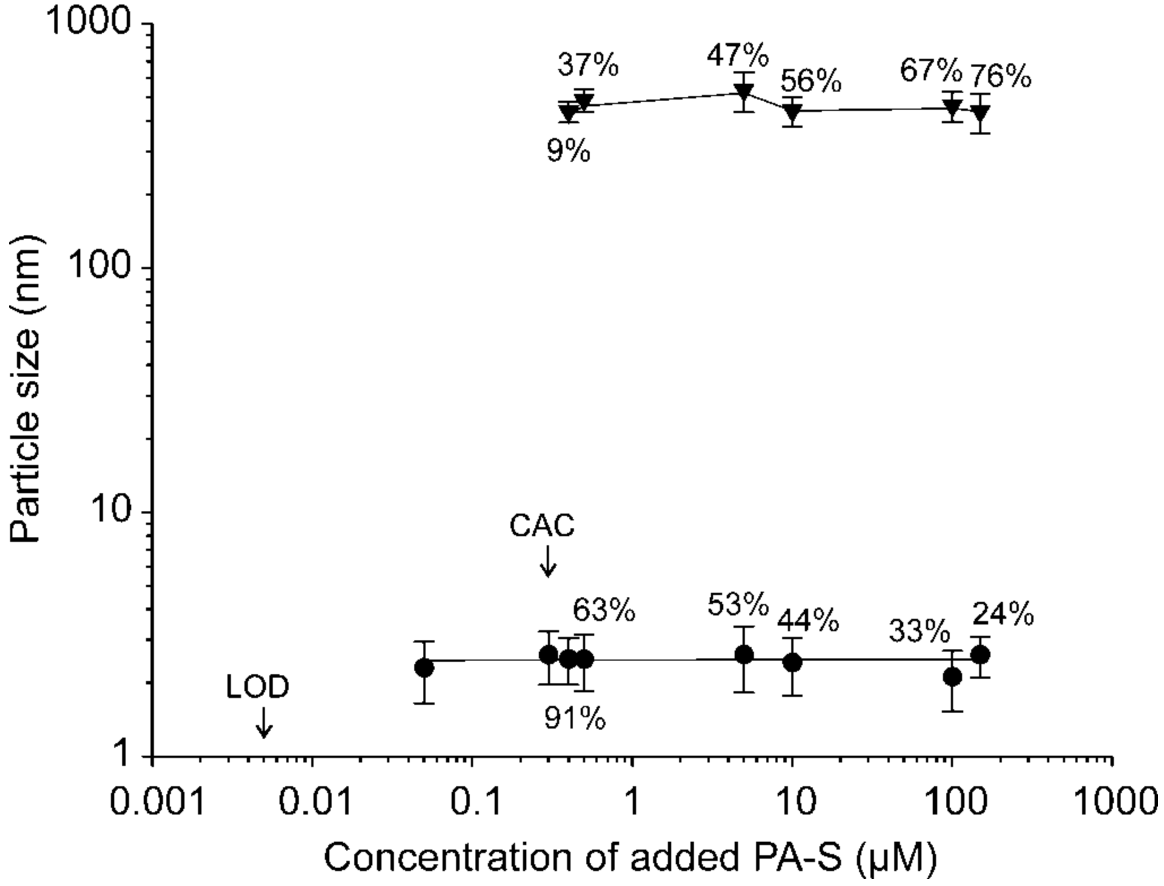

Supplementary Figure 4 | Dependence of particle size on PA-S concentration. Light scattering analysis was used to characterize PA-S (5 nM to 150 µM) in the ECS. At PA-S concentration ≥0.4 µM, two particle sizes were observed. No light scattering was detected at 5 nM (the limit of detection (LOD) provided by the manufacturer was ~1 nm).

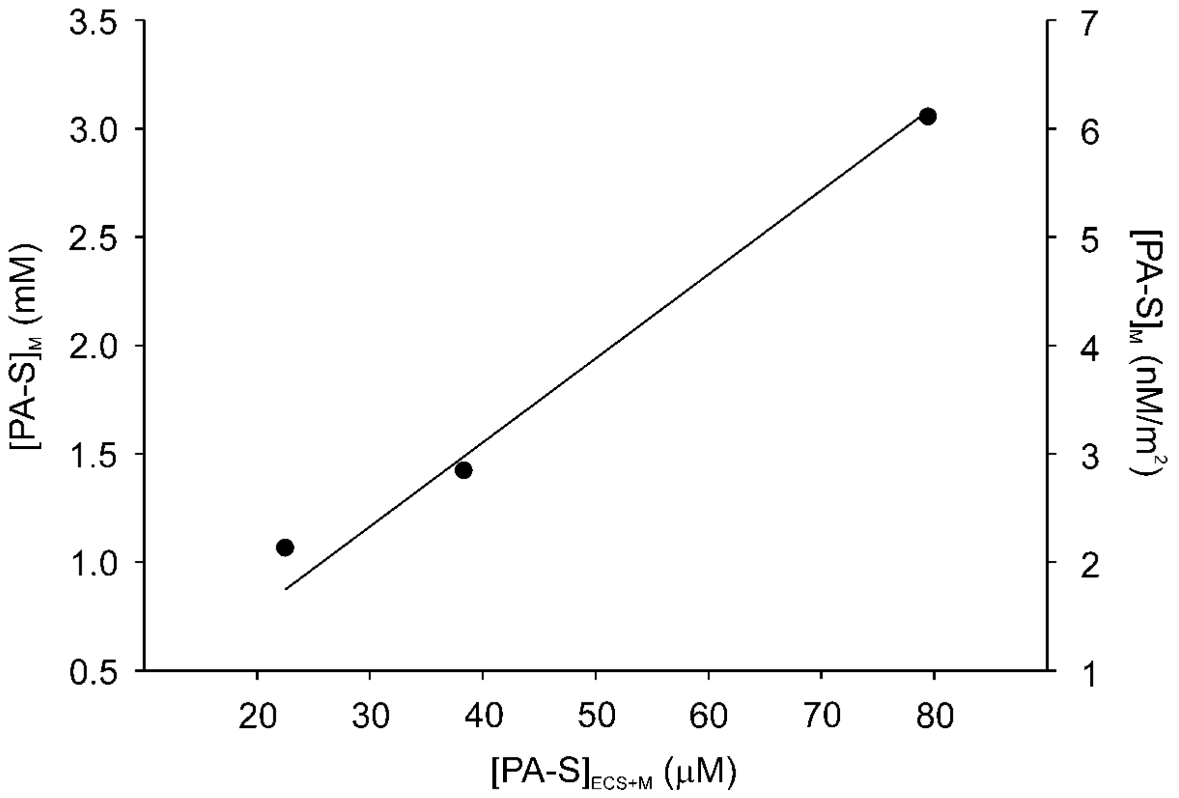

Supplementary Figure 5 | Graph of the partition coefficient of PA-S in the membrane. PA-S concentration was assessed in ECS to which steroid was added from its 20 mM stock solution ($[PA\text{-}S]_{ECS}$) and in the supernatant of a solution containing erythrocyte membranes ($[PA\text{-}S]_{ECS+M}$) (see Methods). The concentration of steroid in the membrane ($[PA\text{-}S]_M$) was calculated from the difference of [PA-S] in the control solution ($[PA\text{-}S]_{ECS}$) and that in the supernatant containing membranes ($[PA\text{-}S]_{ECS+M}$) according to $[PA\text{-}S]_M = ([PA\text{-}S]_{ECS} - [PA\text{-}S]_{ECS+M}) \cdot V_{solution}/V_{membrane}$ (where V denotes volume). Data points were fitted to the linear equation of the following form y = 38.8 * x; The partition coefficient for PA-S was calculated from the slope of the fit; log D = 1.59, which was similar to the calculated log D (1.67 at pH 7.4) determined using the online program CHEMICALIZE (http://www.chemicalize.org/). Data points represent the mean of two independent measurements. The values of the partition coefficient (log D) of PA-S in the erythrocyte cytoplasmic membrane was calculated assuming 136 μm^2 as the mean surface area of a rat erythrocyte and 4 nm as the mean membrane thickness. For the calculation of the PA-S concentration normalized with respect to the membrane surface area, steroid distribution was calculated for the extracellular membrane leaf.

Supplementary Figure 6 | Estimating PA-S monomer concentration near the membrane. Reaction-diffusion modeling was used to estimate PA-S monomer concentration in the close vicinity of the cell membrane. The result is expressed by Eq. S4 and is numerically evaluated in Supplementary Figure 6a. Our model is based on the following assumptions:

i. Near the cell (in the unstirred layer[1,2] with an expected thickness at least several μm), the extracellular fluid is not well mixed, and advection in the direction perpendicular to the membrane is negligible.
ii. The steroid exits the membrane into the aqueous phase predominantly in the monomer form. The steroid enters the membrane predominantly in the aggregate form.
iii. In the aqueous phase, the aggregation of monomers into clusters can be described by diffusion-controlled kinetics.

Under these assumptions, the monomer concentration c(x,t) at a distance x from the membrane satisfies the following reaction-diffusion equation:

$$\frac{\partial c}{\partial t} = D\frac{\partial^2 c}{\partial x^2} - k_\mathrm{a}c^2 + R_\mathrm{d}\,, \qquad \textit{Equation S1}$$

where D is the diffusion constant for the PA-S monomer, k_a is the second-order rate constant for monomer aggregation, and R_d is the rate of monomer dissociation from aggregates. Eq. S1, valid in the aqueous phase, has to be supplemented by boundary conditions. The Fick diffusion flux near the membrane must be equal to the outflux J_out of the monomer from the membrane into the aqueous phase, which gives the boundary condition at the membrane:

$$-D\frac{\partial c}{\partial x}|_{x=0} = k_\mathrm{out}S\,, \qquad \textit{Equation S2}$$

where the outflux J_out was expressed as the product of the rate constant k_out and of the surface density S of the steroid in membrane. Far from the membrane, the concentration decays to low values (comparable to the critical aggregates concentration CAC).

In the steady state, the concentration profile $c(x)$ is independent of time t, and Eq. S1 is readily solved. As will be shown below, the predicted concentration in the vicinity of the membrane significantly exceeds CAC, which implies that the aggregation rate $k_\mathrm{a}c^2$ is greater than the dissociation rate R_d. Neglecting R_d, we obtain the following steady-state profile $c(x)$ satisfying Eqs. E1 and E2:

$$c(x) = \frac{6D}{k_a}\frac{1}{(x+X_0)^2}, \quad \text{where } X_0 = \left(\frac{12D^2}{k_a k_{out} S}\right)^{1/3} \quad . \qquad \textit{Equation S3}$$

The monomer concentration $c(x)$ thus reaches its maximum value c_M in the immediate vicinity of the membrane (i.e., at $x = 0$), and decays to 1/4 of this value at distance $x = X_0$ from the membrane. At distances x significantly exceeding X_0, the rate R_d cannot be neglected in Eq. S1, and Eq. S3 underestimates the monomer concentration. Near the membrane (at distances $x < X_0$), Eq. S3 is adequate. The monomer concentration very near the membrane is thus given by

$$c_M = c(0) = \frac{6}{12^{2/3}}\left(\frac{k_{out}^2 S^2}{k_a D}\right)^{1/3} \quad . \qquad \textit{Equation S4}$$

The equilibrium surface density S was experimentally determined to be $4.32 \cdot 10^{-9}\,\mathrm{M\,m^{-2}}$ (calculated for the PA-S IC_{50} value of 55.7 µM). We estimate the diffusion constant for the PA-S monomer (molecular weight 399) to be $D = 4.65 \cdot 10^{-10}\mathrm{m^2\,sec^{-1}}$ (the value for lactose in water at $25^{\circ}\mathrm{C}$ multiplied by the correcting factor $(342/399)^{1/3} = 0.950$). For the rate constants k_{out} and k_a, only a lower, respectively, upper bound is known. The estimates (see Fig. 5) indicate that $k_{out} \geq 1000\ \mathrm{sec^{-1}}$, while theoretical arguments imply $k_a \leq 7 \cdot 10^9\ \mathrm{M^{-1}\,sec^{-1}} = 7 \cdot 10^6\ \mathrm{M^{-1}m^3sec^{-1}}$ [3]. Using these parameter values in Eq. S4, we obtain a lower bound of $2.05 \cdot 10^{-3}\ \mathrm{M\,m^{-3}} = 2.05$ µM on the monomer concentration c_M very near the membrane. In Supplementary Figure 6a, we plot the predicted values of c_M in the parameter range $1000\ \mathrm{sec^{-1}} \leq k_{out} \leq 2 \cdot 10^4\ \mathrm{sec^{-1}}$ and $10^9\ \mathrm{M^{-1}sec^{-1}} \leq k_a \leq 10^{10}\ \mathrm{M^{-1}sec^{-1}}$. The values of the characteristic decay length X_0 are shown (for the same parameter range) in Supplementary Fig. 6b.

We conclude that at distances less than 0.1 μm from the membrane, the PA-S monomer concentration exceeds CAC by at least one order of magnitude. This significant enhancement of the monomer concentration depends on a reaction-diffusion mechanism in which the membrane acts as the source of monomer. The high outflux of PA-S monomer from the membrane, combined with diffusion and aggregation of the monomer in the extracellular fluid, establishes a steep spatial profile of monomer concentration in the $\sim 1\ \mu$m vicinity of the cell. Consequently, the PA-S monomer concentration reaches values in excess of 2 µM in the immediate vicinity of the membrane.

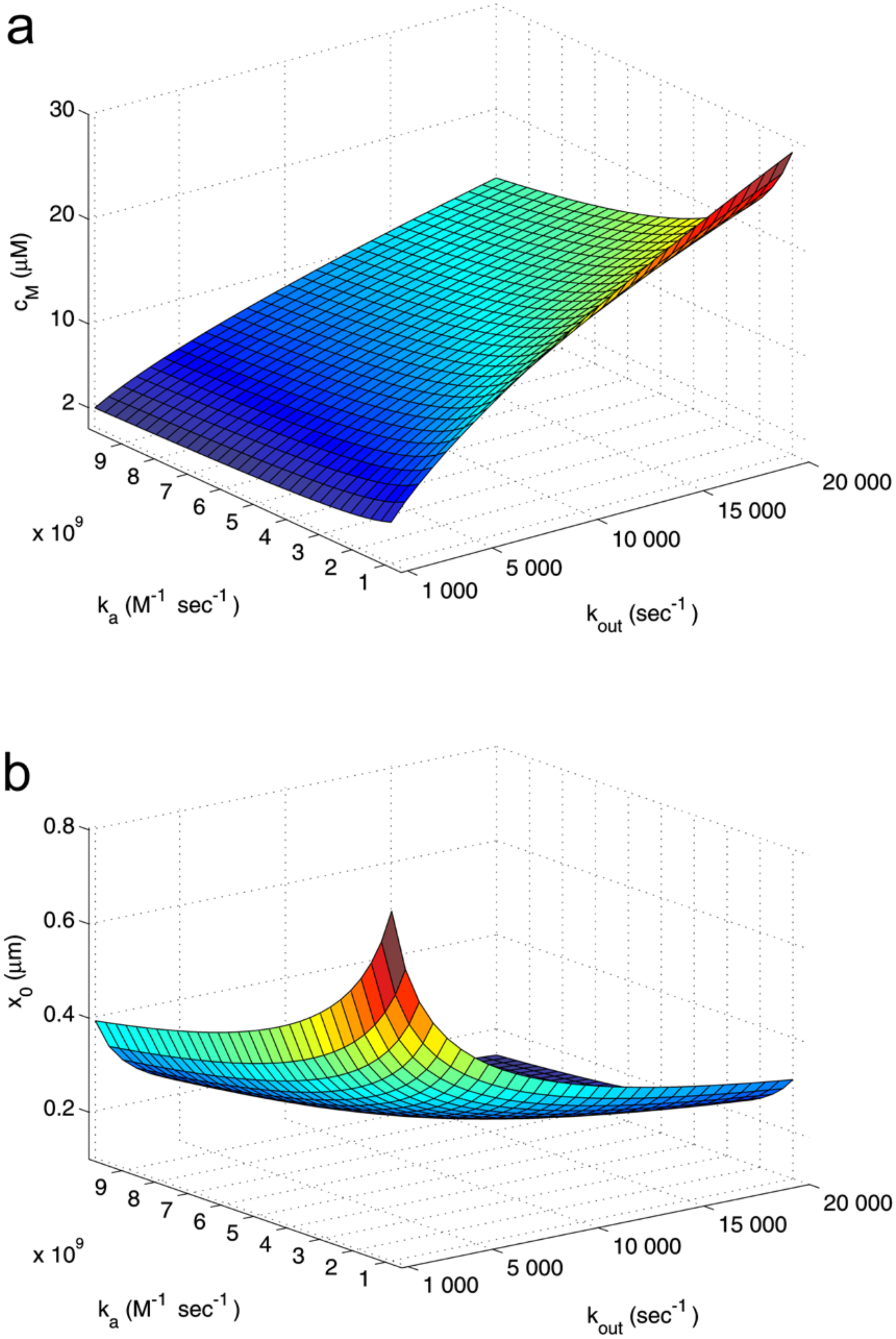
a
30
20
10
2
c_M (μM)
9 8 7 6 5 4 3 2 1
x 10^9
k_a (M^-1 sec^-1)
1 000
5 000
10 000
15 000
20 000
k_out (sec^-1)
b
0.8
0.6
0.4
0.2
x_0 (μm)
9 8 7 6 5 4 3 2 1
x 10^9
k_a (M^-1 sec^-1)
1 000
5 000
10 000
15 000
20 000
k_out (sec^-1)

Dependence of the predicted PA-S monomer concentration profile on the rate constants k_{out} and k_a. (**a**) Monomer concentration c_M in the immediate vicinity of the membrane (as given by Eq. S4.) The membrane surface density of the steroid was assumed to be $4.32 \cdot 10^{-9}$ M m^{-2} (calculated for PA-S IC_{50} value 55.7 µM; see Results). (**b**) Distance X_0 from the membrane at which the monomer concentration decays to 1/4 of c_M (as given by Eq. S3).

References

1. Barry, P.H. & Diamond, J.M. Effects of unstirred layers on membrane phenomena. *Physiol Rev* 64, 763-872 (1984).
2. Pohl, P., Saparov, S.M. & Antonenko, Y.N. The size of the unstirred layer as a function of the solute diffusion coefficient. *Biophys J* 75, 1403-9 (1998).
3. Steinfeld, J.I., Francisco, J.S. & Hase, W.L. Chemical kinetics and dynamics. *Prentice-Hall*, Englewood Cliffs, NJ. (1999).
